# Substrate and Functional Diversity of Protein Lysine Post-translational Modifications

**DOI:** 10.1093/gpbjnl/qzae019

**Published:** 2024-02-28

**Authors:** Bingbing Hao, Kaifeng Chen, Linhui Zhai, Muyin Liu, Bin Liu, Minjia Tan

**Affiliations:** State Key Laboratory of Drug Research, Shanghai Institute of Materia Medica, Chinese Academy of Sciences, Shanghai 201203, China; University of Chinese Academy of Sciences, Beijing 100049, China; Tianjian Laboratory of Advanced Biomedical Sciences, Institute of Advanced Biomedical Sciences, Zhengzhou University, Zhengzhou 450001, China; State Key Laboratory of Drug Research, Shanghai Institute of Materia Medica, Chinese Academy of Sciences, Shanghai 201203, China; University of Chinese Academy of Sciences, Beijing 100049, China; State Key Laboratory of Drug Research, Shanghai Institute of Materia Medica, Chinese Academy of Sciences, Shanghai 201203, China; Zhongshan Institute for Drug Discovery, Shanghai Institute of Materia Medica, Chinese Academy of Sciences, Zhongshan 528400, China; State Key Laboratory of Pharmaceutical Biotechnology, Nanjing University, Nanjing 210023, China; Department of Cardiology, Shanghai Institute of Cardiovascular Diseases, Zhongshan Hospital, Fudan University, Shanghai 200032, China; Jiangsu Key Laboratory of Marine Pharmaceutical Compound Screening, College of Pharmacy, Jiangsu Ocean University, Lianyungang 222005, China; State Key Laboratory of Drug Research, Shanghai Institute of Materia Medica, Chinese Academy of Sciences, Shanghai 201203, China; University of Chinese Academy of Sciences, Beijing 100049, China; Zhongshan Institute for Drug Discovery, Shanghai Institute of Materia Medica, Chinese Academy of Sciences, Zhongshan 528400, China

**Keywords:** Protein lysine PTM, Regulatory enzyme, PTM crosstalk, Drug target, Acylation

## Abstract

Lysine post-translational modifications (PTMs) are widespread and versatile protein PTMs that are involved in diverse biological processes by regulating the fundamental functions of histone and non-histone proteins. Dysregulation of lysine PTMs is implicated in many diseases, and targeting lysine PTM regulatory factors, including writers, erasers, and readers, has become an effective strategy for disease therapy. The continuing development of mass spectrometry (MS) technologies coupled with antibody-based affinity enrichment technologies greatly promotes the discovery and decoding of PTMs. The global characterization of lysine PTMs is crucial for deciphering the regulatory networks, molecular functions, and mechanisms of action of lysine PTMs. In this review, we focus on lysine PTMs, and provide a summary of the regulatory enzymes of diverse lysine PTMs and the proteomics advances in lysine PTMs by MS technologies. We also discuss the types and biological functions of lysine PTM crosstalks on histone and non-histone proteins and current druggable targets of lysine PTM regulatory factors for disease therapy.

## Introduction

Protein post-translational modifications (PTMs) of the epsilon amine group at lysine residues, also known as lysine PTMs, are widespread and versatile protein PTMs that play crucial roles in diverse biological processes, including transcription regulation, signaling transduction, protein degradation, and cell metabolism [1–3]. Lysine PTMs greatly expand the biological functions of histone and non-histone proteins, and dysregulation of lysine PTMs is implicated in various diseases, such as cancer and metabolic diseases [4,5]. Targeting lysine PTM regulatory factors, including writers, erasers, and readers, has been demonstrated to be an effective therapeutic strategy for various diseases [6–11].

The global characterization of lysine PTMs is crucial for understanding their biological functions and regulatory mechanisms in different physiological and pathological states. The continuing development of mass spectrometry (MS) technologies coupled with antibody-based affinity enrichment technologies has greatly promoted the discovery and decoding of PTMs [12,13]. Currently, over 30 types of lysine PTMs have been characterized, and about 300,000 lysine PTM sites have been identified [14]. Accordingly, by system-wide characterization of lysine PTMs on histone and non-histone proteins, the regulatory networks, molecular functions, mechanisms of action, and druggable targets of lysine PTMs have been increasingly deciphered. We recently summarized the proteomics-based characterization of PTMs in drug discovery [15].

In this review, we focus on lysine PTMs, and provide a comprehensive summary of the regulatory enzymes of diverse lysine PTMs and the proteomics advances in lysine PTMs based on MS technologies. We discuss the types and biological functions of crosstalks between lysine PTMs on both histone and non-histone proteins, as well as the current druggable targets of lysine PTM regulatory factors for disease therapy.

## Histone lysine PTMs and regulatory factors

### Methylation

Histone lysine methylation (Kme) types include mono-methylation (Kme1), di-methylation (Kme2), and tri-methylation (Kme3) [16], which are important epigenetic markers and dynamically regulated by methyltransferases (writers) and demethylases (erasers). Histone Kme plays active or repressive transcriptional functions dependent on distinct effector proteins (readers). For example, the methylation of H3K4, H3K36, and H3K79 promotes transcription, while the methylation of H3K9, H3K27, and H4K20 represses transcription. The functions of different Kme sites and their corresponding regulatory enzymes (writers, readers, and erasers) have been extensively reviewed in previous reports [17,18]. In this section, we emphasize the newly identified regulators of Kme (Table S1).

A series of H3K27me3-binding proteins that mediate gene repression have been identified in recent years. H3K27me3 is a well-known transcriptionally repressive histone marker [19], catalyzed by polycomb repressive complex 2 (PRC2) and recognized by PRC1, which further catalyzes H2A ubiquitination and promotes chromatin compaction. This interplay between PRC2 and PRC1 is a classical mechanism which suppresses transcription in mammals and plants [20]. However, in fungi, PRC1 is absent, and the mechanism by which H3K27me3 represses transcription is unclear. In 2020, a novel H3K27me3-binding protein, effector of polycomb repression 1 (EPR-1), was discovered in the filamentous fungus *Neurospora crassa.* EPR-1 recognized H3K27me3 through a bromo-adjacent homology (BAH) domain and modulated gene silencing through a plant homeodomain (PHD) [21]. Moreover, homologs of EPR-1 were present in other species, indicating an alternate polycomb repression pathway. In the same year, BAH domain-containing protein AIPP3 and PHD domain-containing proteins AIPP2 and PAIPP2 in *Arabidopsis* were reported as H3K27me3 readers [22]. They recruited CPL2, a Pol II carboxyl terminal domain phosphatase, to repress Pol II release from transcriptional start sites. A BAH domain-containing H3K27me3 reader was also reported in mammals. BAH and coiled-coil domain-containing protein 1 (BAHCC1) is bound to H3K27me3-marked genes and interacts with transcriptional corepressors, such as SAP30-binding protein (SAP30BP) and histone deacetylase (HDAC) [23]. These newly identified H3K27me3 readers uncover novel mechanisms of H3K27me3-mediated gene repression beyond interplay of PRC1 and PRC2.

### Ubiquitination

Lysine ubiquitination (Kub) entails the covalent attachment of mono- or poly- ubiquitin, which is a protein of 76 amino acids. Kub types of histones are generally mono-ubiquitinated, such as H2AK13/15ub, H2AK118/119ub, H2AK125/127/129ub, and H2BK120ub [24,25]. Histone ubiquitination is catalyzed by sequential actions of E1, E2, and E3 enzymes, removed by deubiquitinating enzymes (DUBs), and recognized by diverse readers to carry out biological functions [26]. The regulatory enzymes and functions of histone ubiquitination have been systematically summarized in previous reviews [26], and thus in this section we highlight some newly identified regulators and their roles in ubiquitination-mediated chromatin regulation (Table S1).

The ubiquitination of H2AK13/15 and H2AK125/127/129 often occurs in DNA damage repair (DDR). At DNA double-strand breaks (DSBs), the E3 ligase RNF168 catalyzes H2AK13/15 mono-ubiquitination and recruits 53BP1 for non-homologous end joining (NHEJ) DSB repair, while the BRCA1–BRAD1 E3 ligase complex catalyzes H2AK125/127/129 mono-ubiquitination and mediates homologous recombination (HR) [26]. However, how histone controls the selection of HR or NHEJ pathways remains unclear. In recent years, BRAD1 in the BRCA1–BRAD1 E3 complex was identified as a new H2AK15ub reader [27–29]. BRAD1 recognized H2AK15ub and unmethylated H4K20me1/2, recruited BRCA1, and triggered HR. Conversely, when H4K20 was methylated, H2AK15ub and H4K20me1/2 were recognized by 53BP1, and NHEJ was triggered. These findings uncover how histone modification states regulate the equilibrium of two DDR pathways.

The ubiquitination of H2AK118/119 and H2BK120 is mainly modulated during transcriptional regulation. H2AK118/119ub is catalyzed by PRC1 [30], which recruits the PRC2 cofactors AEBP2 and JARID2 to facilitate H3K27me3 [31]. H2AK118/119ub can also recruit RYBP–PRC1 or YAF2–PRC1, two PRC1 variants, and promote H2AK119ub in a positive-feedback model [32]. Recent reports showed that DNA methyltransferase 3a1 (DNMT3A1) was an essential factor in neurodevelopmental gene expression, and recognized H2AK119ub through a disordered N-terminal domain, further linking H2AK119ub with DNA methylation [33,34]. H2BK120ub is known to recruit histone methyltransferases, disruptor of telomeric silencing 1 like (DOT1L) and COMPASS, to promote H3K79 and H3K4 methylation. In 2019, a structural study revealed how H2BK120ub interacted with and stimulated DOT1L by restricting it on the face of the nucleosome, uncovering the mechanism by which H2BK120ub regulated H3K79 methylation [35]. Recent studies also indicated that H2BK120ub influenced H3K4 methylation beyond recruiting COMPASS [36]. It was reported that proteasomal subunit RPT6 recognized H2BK120ub and promoted H3K4me3 in a non-canonical manner. Moreover, the removal of H2BK120ub by USP38 can recruit demethylase KDM5B and decreased H3K4me3 [37].

### Acylation

**Figure 1:**
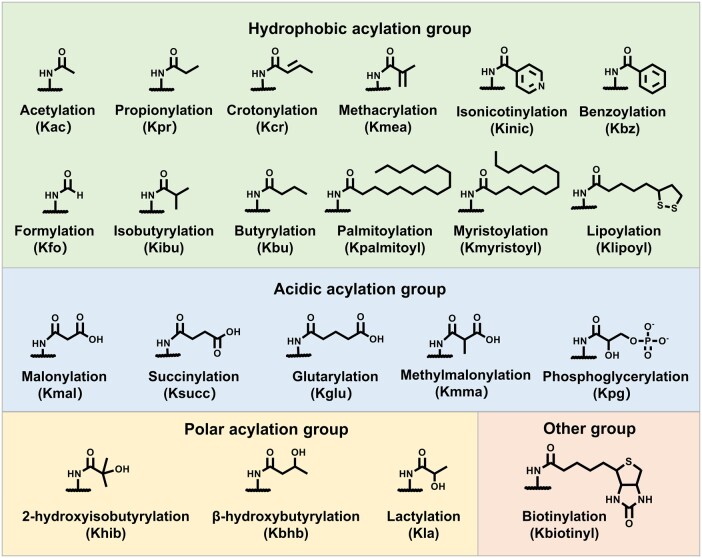
Structures of lysine acylation

**Figure 2:**
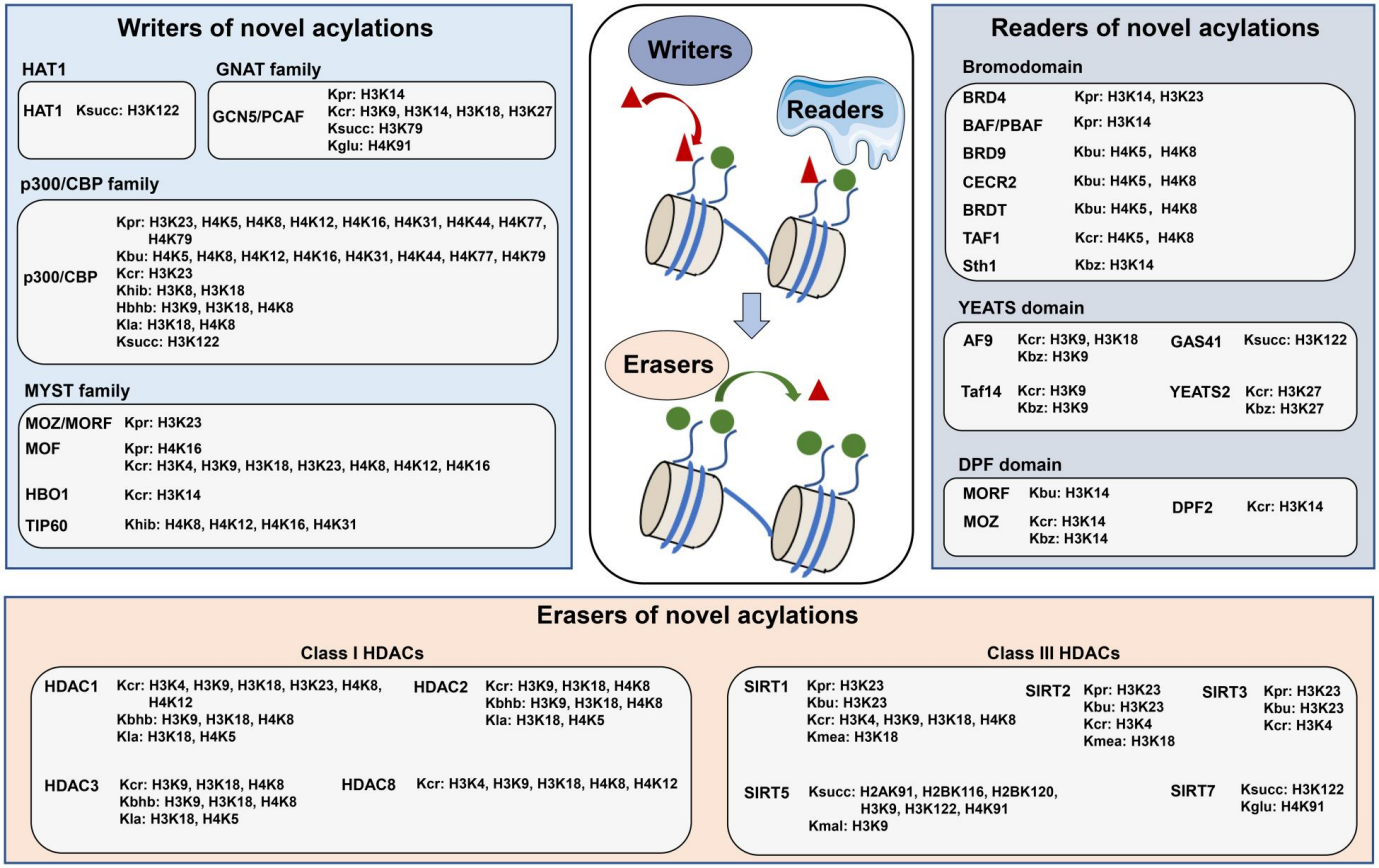
Writers, readers, and erasers of novel histone acylations Writers that catalyze the addition include HAT1, GNAT family, p300/CBP family, and MYST family. Readers that recognize novel acylations include bromodomain-, YEATS domain-, and DPF domain-containing proteins. Erasers that catalyze the removal include Class I and Class III HDACs. These regulators are listed with their regulated modification types and sites. Red triangles and green circles represent different histone acylations. HAT, histone acetyltransferase; GNAT, GCN5-related *N*-acetyltransferase; GCN5, general control non-depressible 5; PCAF, p300/CBP-associated factor; MOZ, monocytic leukemic zinc finger; MORF, MOZ-related factor; MOF, males absent on the first; HBO1, HAT binding to origin recognition complex 1; BRD, bromodomain-containing protein; BAF, BRG1/BRM-associated factor; PBAF, polybromo-associated BAF; CECR2, cat eye syndrome chromosome region candidate 2; BRDT, bromodomain testis-specific protein; Taf, transcription initiation factor TFIID subunit; AF9, ALL1-fused gene from chromosome 9 protein; GAS41, glioma-amplified sequence 41; YEATS2, YEATS domain-containing protein 2; DPF, double PHD finger; HDAC, histone deacetylase; SIRT, sirtuin.

Protein lysine acylation is an important type of protein PTMs, in which different acyl groups covalently bind to lysine residues in proteins (**Figure 1**). Among them, lysine acetylation (Kac) was reported as the first histone acylation in 1963 and was well studied as a positive epigenetic marker [38,39]. Up to 40 Kac sites are found on core histones and regulated by 22 histone acetyltransferases (HATs) and 18 HDACs [3]. They are recognized by bromodomain-containing proteins and further activate transcription. In recent years, a series of novel histone lysine acylation types with diverse structures and different functions were discovered, such as propionylation (Kpr), crotonylation (Kcr), lactylation (Kla), and succinylation (Ksucc) [2]. Depending on the chemical properties of these acylation groups, they are generally clustered into three groups: hydrophobic, polar, and acidic groups. How these novel acylations are regulated is of great interest to researchers. In this section, we focus on writers, erasers, and readers of acylations (**Figure 2**; Table S1).

#### Hydrophobic acylation group

Hydrophobic lysine acylations include Kac, Kpr [40], butyrylation (Kbu) [41], Kcr [42], methacrylation (Kmea) [43], isobutyrylation (Kibu) [44], benzoylation (Kbz) [45], and isonicotinylation (Kinic) [46]. Despite containing diverse hydrocarbon chains, they all neutralize a positive charge and increase the hydrophobicity and size of lysine residues.

Kpr and Kbu were first reported as novel histone acylations in 2007 [41]. Their donors were derived from endogenous metabolites: propionyl-coenzyme A (CoA) was from odd-chain fatty acid and amino acid catabolism, and butyryl-CoA was from oxidation of fatty acids [47]. p300/CBP was first discovered as a Kpr and Kbu writer [48]. Later, general control non-depressible 5 (GCN5), p300/CBP-associated factor (PCAF) in the GCN5-related *N*-acetyltransferase (GNAT) family, and males absent on the first (MOF), monocytic leukemic zinc finger (MOZ), and HAT binding to origin recognition complex 1 (HBO1) in the MYST (Moz, Ybf2/Sas3, Sas2, Tip60) family were also found to have propionyltransferase activity [49–51]. However, butyryltransferases have barely been reported. Previous reports showed that the removal of both Kpr and Kbu was catalyzed by sirtuin 1–3 (SIRT1–3) [52]. Kpr and Kbu recruit readers, such as bromodomain-containing proteins, to exercise transcriptional regulatory functions. An early study revealed that bromodomain-containing protein 4 (BRD4) recognized Kpr and Kbu, but much more weakly than Kac [53]. In recent years, H3K14pr was reported to interact with BRG1/BRM-associated factor (BAF) complex and stimulate transcription [54], and Kbu was reported to compete with Kac at H4K5 and H4K8 and regulate bromodomain testis-specific protein (BRDT) binding [55]. Structural studies also discovered a subset of bromodomain-containing proteins that recognized Kbu [56].

Kcr, which was first discovered in 2011 [42], contains a four-carbon chain with C–C π bond which leads to a unique rigid planar conformation. The donor crotonyl-CoA is abundant intermediate in butyryl-CoA and acetyl-CoA metabolic pathways and is also directly synthesized from crotonate. A series of writers were identified that catalyze Kcr including p300/CBP [57], GCN5[58], PCAF in the GNAT family, and MOF in the MYST family [59]. The removal of Kcr was performed by Zn^2+^-dependent HDAC1/2/3/8 and NAD^+^-dependent SIRT1/2/3 [60–63]. Due to the distinct conformation, Kcr could be identified by YEATS (Yaf9, ENL, AF9, Taf14, Sas5) and double PHD finger (DPF) domain-containing proteins with even higher affinities than Kac. The YEATS domain was identified as a Kac reader in 2014 and was also reported to prefer Kcr over Kac because of the π-aromatic interaction between the C–C π bond in Kcr with F59 and Y78 in the YEATS binding pocket [64]. In the YEATS family, AF9 interacted with H3K9cr and H3K18cr and positively regulated gene expression [64], while Taf14 recognized H3K9cr and mediated gene suppression [65]. The DPF domain preferred Kcr because a hydrophobic “dead-end” pocket [66] and DPF-containing proteins such as MOZ and MOZ-related factor (MORF) were reported as a reader that recognized H3K14cr.

In addition to Kcr, two short-chain hydrophobic acylations, Kibu and Kmea were also discovered in recent years [43,44]. Kibu is a structural isomer of Kbu. The donor isobutyryl-CoA is a metabolite of valine catabolism and branched chain fatty acid oxidation and is further catalyzed by p300 and HAT1 to form Kibu. However, only two histone Kibu sites were identified, H3K14ibu and H3K23ibu, and the function of Kibu is largely unknown. Kmea is a structural isomer of Kcr. It was proposed to be derived from methacrylate, which is produced in mitochondria by valine catabolism and is dynamically regulated by HAT1 and SIRT2. However, the epigenetic function of Kmea remains largely unclear.

Apart from short-chain hydrophobic acylations, aromatic group-containing and pyridine ring-containing acylations were also discovered in recent years. Kbz was the first reported histone lysine modification with an aromatic side chain [45]. A possible precursor of Kbz, benzoyl-CoA, is mainly derived from sodium benzoate, which is widely used as a food preservative. Kbz can be added by GCN5 and removed by SIRT2 [67]. A series of readers were reported to recognize Kbz [68]. For example, DPF domain-containing MOZ recognized H3K14bz, YEATS domain-containing AF9 recognized H3K9bz, and YEATS domain-containing 2 (YEATS2) recognized H3K27bz. These variable reader proteins indicate the epigenetic regulatory role of Kbz. Kinic is a pyridine ring-containing lysine modification reported in 2021 [46]. Isoniazid, a widely used first-line anti-tuberculosis drug, was proposed to promote the synthesis of isonicotinly-CoA and improved Kinic. Kinic was found to be regulated by p300/CBP and HDAC3, and was reported to regulate gene expression involved in tumorigenesis. The discovery of exogenously derived histone modifications, such as Kbz and Kinic, raises new concerns about the mechanism of action as well as safety of preservatives and drugs. However, the endogenous metabolism of precursors of Kbz and Kinic remains largely unknown.

#### Polar acylation group

Polar acylations include 2-hydroxyisobutyrylation (Khib) [69], β-hydroxybutyrylation (Kbhb) [70], and Kla [71]. They all contain a hydroxylated side chain to allow the formation of hydrogen bonds between modified lysine and other molecules.

Khib was discovered in 2014 as the first polar acylation [69]. A possible Khib donor is 2-hydroxyisobutyryl-CoA, which is an intermediate in some gut microbiota and a detectable organic acid associated with lactic acidosis. p300 and the MYST family member TIP60 catalyze Khib, while HDAC1/2/3 can remove Khib [72–74]. The reader of Khib has not been reported yet. Kbhb is a structural isomer of Khib and was reported in 2016 [70]. β-hydroxybutyrate, a key element of the ketogenic diet, was proposed to serve as a precursor of Kbhb. p300 and HDAC1/2/3 were reported to dynamically regulate Kbhb [75]. Kbhb can be induced by starvation, such as H3K9bhb, and mark metabolic-related genes [70].

The discovery of Kla in 2019 provided new insight into the link between glucose metabolism and histone modifications [71]. Currently, only p300 has been identified as a Kla writer. A systematic screen revealed that HDAC1/2/3 were lysine delactylases, among which HDAC3 was the most efficient one [76]. Kla was reported to mediate gene expression, such as wound healing-related genes in macrophages. Up to date, there are only rare reports of Kla readers, and the molecular mechanisms of its gene regulatory effects remain to be explored.

#### Acidic acylation group

Acidic acylations include malonylation (Kmal) [77], Ksucc [77], and glutarylation (Kglu) [78]. They convert lysine residues from a positive charge state to a negative charge state, which strongly impairs the interaction between histones and DNA.

Ksucc, Kmal, and Kglu were all proposed to be derived from intercellular intermediates. Ksucc was associated with the tricarboxylic acid (TCA) cycle and branched chain amino acid metabolism [79]. Kmal could be derived from acetyl-CoA and β-oxidation of odd-chain-length dicarboxylic acids [80]. Kglu was associated with lysine and tryptophan metabolism [78]. GCN5 was reported to catalyze Ksucc and Kglu on histones. GCN5 coupled with metabolic enzymes regulates corresponding histone acylation. For example, GCN5 together with the α-ketoglutarate dehydrogenase (α-KGDH) complex regulates H3K79succ [81]. Similarly, GCN5 coupled with α-ketoadipate dehydrogenase (α-KADH) regulates H4K91glu [82]. Recently, a study suggests that GCN5 rarely exhibits Ksucc and Kglu activities, and the reported histone Ksucc and Kglu sites catalyzed by GCN5 might result from nonenzymatic acylation [83]. Removal of Ksucc, Kmal, and Kglu is catalyzed by SIRT5, which structurally prefers negatively charged acyl groups [84]. In recent years, SIRT7 has also been reported to be a desuccinylase and deglutarylase [85]. However, readers of these acidic acylations are rarely reported. Up to date, only the YEATS domain-containing GAS41 was identified to recognize Ksucc, but its epigenetic function remains to be explored [86].

## Proteomic characterization of non-histone lysine PTMs

### Methylation

Kme occurs not only on histones but also on thousands of non-histone substrates [16]. Non-histone methylation plays an important role in regulating protein–protein interactions, protein stability, and subcellular localization, but systematic mapping of the lysine methylome remains a challenge [18]. Despite recent advantages of MS-based proteomics in the study of the acetylome, phosphoproteome, and ubiquitinome, the application of MS-based proteomics in the methylome is hampered by inefficient enrichment of Kme.

**Figure 3:**
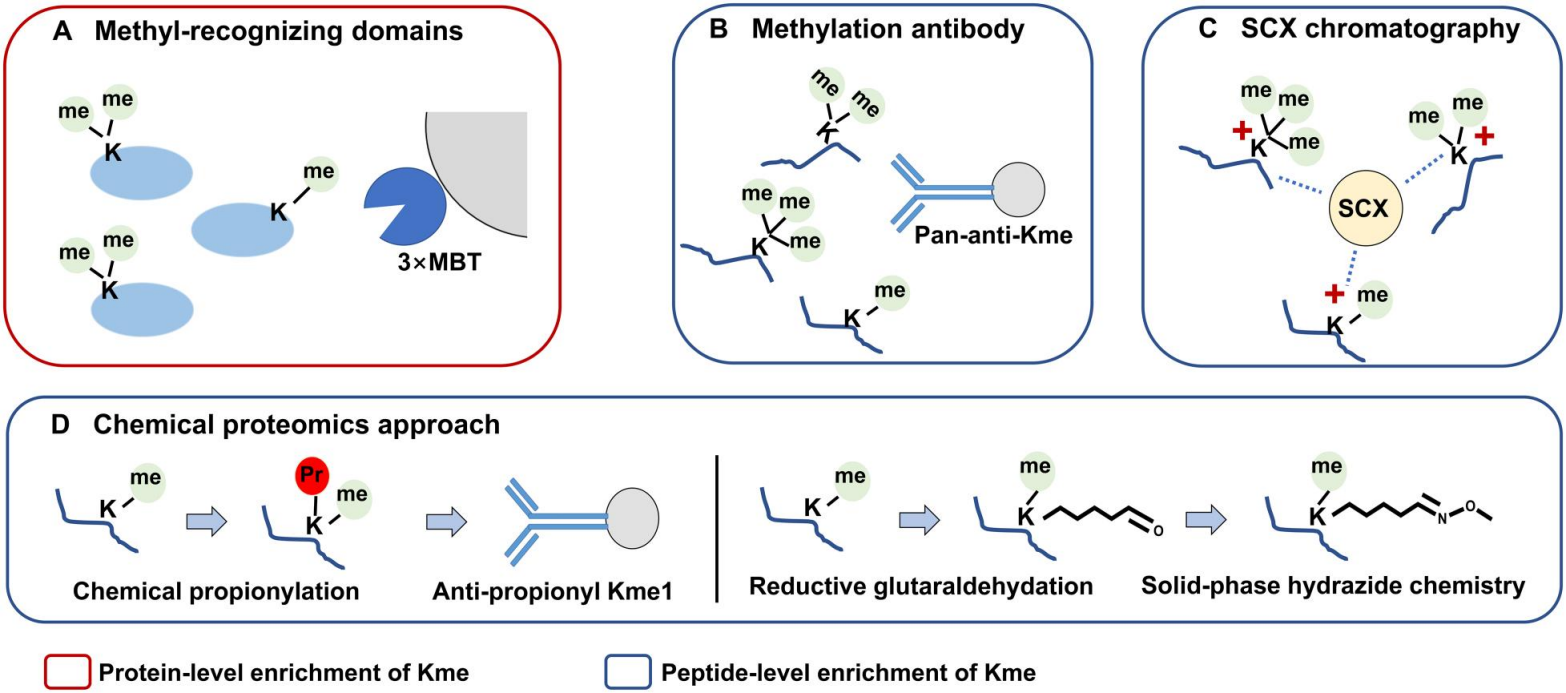
Lysine methylation enrichment strategies for LC-MS/MS analysis **A**. Protein-level enrichment includes methyl-recognizing domain-based strategy. **B**.–**D**. Peptide-level enrichment includes pan-anti-methylation antibody-based strategy (B), SCX-based chromatography (C), and chemical proteomics approach (D). LC, liquid chromatography; MS, mass spectrometry; SCX, strong cation-exchange.

Scott et al. reported a method to enrich methylated proteins with methyl-recognizing domains [87]. They applied triple malignant brain tumor domains (3×MBT) of Lethal(3)malignant brain tumor-like protein 1 (L3MBTL1) to bind Kme1 and Kme2 and improved MS-based identification of methylated proteins (**Figure 3**A). However, identifying Kme sites directly remains challenging. Antibody-based peptide-level enrichment is widely used in MS-based proteomics analysis of PTMs (Figure 3B). A pan-antibody against Kme1, Kme2, and Kme3 was reported in 2013 by Cao and his colleagues [88]. They combined immunoprecipitation, strong cation-exchange (SCX) (Figure 3C), and liquid chromatography-tandem mass spectrometry (LC-MS/MS) analysis to achieve the identification of 552 Kme sites on 413 proteins, providing the most comprehensive methylome dataset at that time. Later in 2016, the same group further optimized the enrichment method for methylated peptides by introducing prefractionation before enrichment and achieved the identification of 1246 Kme1, 59 Kme2, and 53 Kme3 sites in one experiment [89]. Combined with a quantitative approach, this procedure was also used to quantitatively analyze Kme sites across the proteome. For example, they used a stable isotope labeling by amino acids in cell culture (SILAC)-based quantitative methylome to study the activity of the lysine methyltransferase SMYD2 [90]. This study identified 1032 Kme1 sites and quantified 273 of them in esophageal squamous cell carcinoma (ESCC) and identified 1861 Kme1 sites and quantified 664 of them in SMYD2-overexpressed ESCC. Thirty-five Kme1 sites were both down-regulated by *SMYD2* knockdown or inhibition, and four proteins were confirmed as novel SMYD2 substrates. A pan-antibody-based methylome study was also applied in plants [91]. Liang et al. provided a comprehensive global survey of Kme in *Arabidopsis*, in which they identified 263 proteins harboring 381 Kme sites. These Kme sites in *Arabidopsis* were mainly involved in protein complex assembly, RNA processing, and metabolic processes.

In addition to the pan-antibody against Kme, researchers have also developed chemical proteomics approach for Kme1 profiling (Figure 3D). Our previous study introduced chemical propionylation of Kme1 followed by enrichment using anti-propionyl Kme1 antibody and identified 446 Kme1 sites on 398 proteins [92]. In 2020, we combined chemical propionylation-based Kme1 enrichment and pan-antibody-based Kme2 and Kme3 enrichment to study lysine methylome features in wild-type and mutant Enhancer of zeste homolog 2 (*EZH2*) cancer cells [93]. This study identified 122 Kme1 sites, 52 Kme2 sites, and 38 Kme3 sites with 86 Kme1, 5 Kme2, and 26 Kme3 sites quantified. This study underscored the role of Kme substrates and potential crosstalk with other PTMs in hematological cancers with aberrant EZH2 expression.

An antibody-free method was also developed for Kme1 enrichment. In 2022, Li et al. carried out reductive glutaraldehydation on Kme1 followed by solid-phase hydrazide enrichment, and identified a comparable number of Kme1 sites with much less starting material [94] (Figure 3D), providing the first antibody-free approach for Kme1 enrichment as a potential method for methylome studies. A chromatographic approach is also a feasible method for enriching methylated peptides as tryptic methyl-peptides are always positively charged. However, due to the interference of histidine-containing peptides, the performance of SCX in Kme peptide enrichment is not satisfactory. In 2019, Wang et al. presented multidimensional tandem chromatography for methylome analysis [95]. After enrichment by SCX, they applied immobilized metal ion affinity chromatography (IMAC) to remove histidine-containing peptides and high-pH reverse-phase chromatography (RPC) to reduce sample complexity. The combination of SCX, IMAC, and high-pH RPC achieved the identification of 860 methylation sites, and 27.21% of them were Kme sites. Researchers also utilized chemical strategies to deplete histidine-containing peptides [96]. Imidazole carbonylation of histidine for capture by hydrazide resin enabled almost complete depletion of histidine-containing peptides. This approach further improved the detection of low-abundance Kme sites.

### Ubiquitin-like modifications

#### Ubiquitination

**Figure 4:**
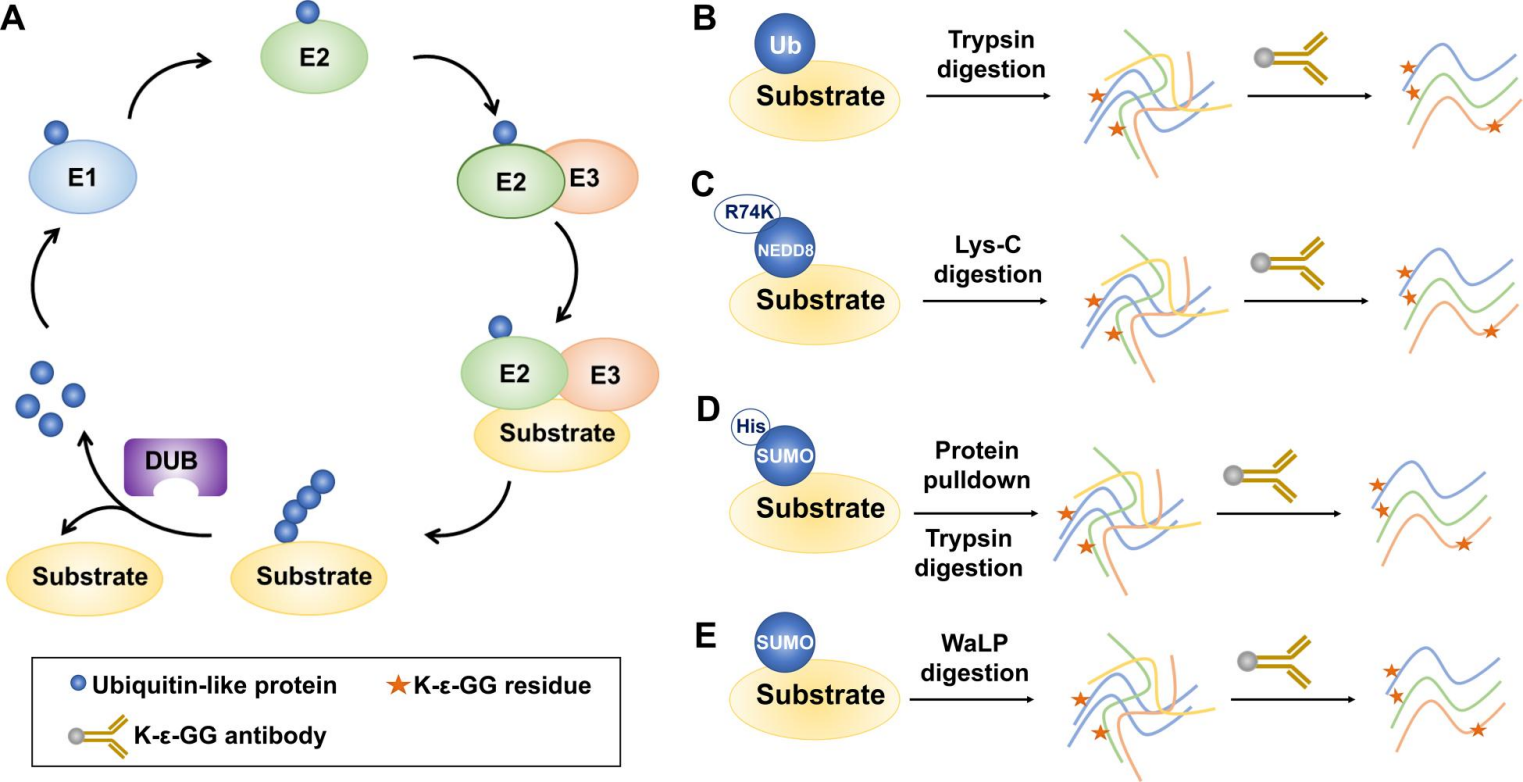
The catalytic process and MS-based identification strategies of ubiquitin-like modifications **A**. The catalytic process of ubiquitin-like modifications by E1/E2/E3 enzymes and de-modified enzymes. **B**.–**E**. The MS-based identification strategies of ubiquitination (B), neddylation (C), and sumoylation (D and E). DUB, deubiquitinating enzyme; Ub, ubiquitin; NEDD8, neural precursor cell expressed developmentally downregulated protein 8; SUMO, small ubiquitin-like modifier; WaLP, wild-type α-lytic protease.

Kub on non-histone proteins is also catalyzed by E1/E2/E3 enzymes and reversibly removed by DUBs (**Figure 4**A), and is mainly involved in protein turnover and signaling transduction. The delineation of the Kub signals is crucial for understanding the roles of Kub in cellular processes.

Ubiquitin attached to substrates leave a diglycine (-GG-) group on the side chain of lysine residues (K-ε-GG) on substrates after trypsin digestion, which facilitates the identification of Kub sites across the proteome by an MS-based proteomics approach. In 2003, the first report using this approach mapped 110 Kub sites in yeast by LC-MS analysis [97]. Applying similar methods, 294 Kub sites in response to a protease inhibitor and 44 Kub sites under control conditions were identified in the mammalian cells [98,99]. The emergence of the K-ε-GG monoclonal antibody accelerated the identification of Kub sites, which enabled more ubiquitinated peptides to be identified in mammalian cells and tissues by combining MS and ubiquitin remnant immunoaffinity enrichment strategies [100–102] (Figure 4B). By combining a SILAC-based quantitative proteomics approach, the roles of Kub in specific cellular pathways, including protein turnover regulation [103], DNA damage response [104], inflammatory response [105], TNF signaling [106], and CRP-XL signaling [107], were explored at the proteome-wide level of Kub sites. In addition, the substrates of Kub regulatory enzymes including E3 ligases and DUBs were also systemically identified by a similar approach [108–114]. Previous reports also indicated that the proteome-wide mapping of Kub sites by quantitative proteomics was critical for discovering the mechanisms of action of drugs, especially ubiquitination-related drugs, proteasome inhibitors, and protein-targeted degradation drugs [115–117]. However, the K-ε-GG epitope is also generated in other PTMs, such as neddylation, after trypsin digestion, which leads to confusion of different PTM sites by this approach. Thus, Akimov et al. developed a solution to this problem by generating an antibody that recognized 13 remnant amino acids of ubiquitin after Lys-C digestion, which enabled the identification of over 63,000 Kub sites under proteasome inhibitor treatment [103].

Combining antibody-based affinity enrichment technology with tandem mass tag (TMT)-based quantitative proteomics approach allows quantitative analysis of Kub sites under more conditions and even at the tissue level. In 2016, Rose et al. simultaneously compared changes in Kub sites under 10 conditions, which led to the quantitation of 5000–9000 Kub events during mitophagy [118]. In addition, a highly sensitive, rapid, and multiplexed method termed UbiFast was developed to monitor the changes of Kub sites, which rediscovered substrates of the E3 ligase targeting drug lenalidomide [119]. Notably, this approach was also applied to decipher the code of Kub in clinical tumor tissues such as lung squamous cell carcinoma [120].

#### Neddylation

Neddylation is a ubiquitin-like modification in which neural precursor cell expressed developmentally downregulated protein 8 (NEDD8) is transferred to substrates via multi-enzyme cascade catalysis. Neddylation is involved in several cellular processes, such as protein turnover, signal transduction, and transcriptional regulation [121,122]. The first systematic study of neddylated substrates identified 496 potential neddylated proteins related to transcription, DNA repair and replication, cell cycle regulation, and chromatin organization by coupling exogenous overexpression of NEDD8, affinity enrichment, and MS technology [123]. This study identified the neddylation sites of the best-characterized neddylated substrates, cullins [123]. In addition, other studies also identified more potential neddylated substrates involved in stress granule (SG) assembly, proteotoxic stress, and cholangiocarcinoma using the similar approach [124–126]. However, these studies only identified the neddylated substrates at the protein level rather than at the amino acid resolution. This is because the remnant residue K-ε-GG of neddylation after trypsin digestion, required for K-ε-GG antibody enrichment of neddylated peptides, is the same as that of Kub, and overexpression of exogenous tagged NEDD8 affects the level of Kub in the proteome. Thus, it is difficult to distinguish the genuine neddylated substrates at the site resolution. Recently, Vogl et al. developed a serial NEDD8-ubiquitin substrate profiling technology based on a mutation at R74 (R74K) in the endogenous NEDD8 and immunoaffinity purification (Figure 4C), which identified 607 putative neddylation sites on 341 proteins [127]. Furthermore, by this approach, Lobato-Gil et al. identified 1101 unique neddylation sites in 620 proteins, which also uncovered canonical and atypical neddylated substrate proteins [128]. Nevertheless, there is currently no valid method to realize the identification and quantification of neddylated substrates at the resolution of modified sites across the proteome at the tissue level.

#### Sumoylation

Small ubiquitin-like modifiers (SUMOs) are reversibly conjugated to lysine residues of protein substrates, which form an important PTM, sumoylation, involved in DNA damage, gene transcription, RNA splicing, and protein degradation [129–131]. SUMOs are evolutionarily conserved proteins, and the dominant SUMOs are SUMO1, SUMO2, and SUMO3 in mammalian cells. Similar to Kub and neddylation, SUMOs are also step-by-step catalyzed by E1, E2, and E3 enzymes. However, distinct from Kub and neddylation, sumoylation generates a longer sequence (19 or 32 amino acids) after trypsin digestion, which hampers the system-wide identification of sumoylation across the proteome because of the limitation of MS identification. Nevertheless, several approaches are still being developed to map sumoylation sites. Currently, more than 70,000 sumoylation sites on about 7500 proteins have been identified according to the Compendium of Protein Lysine Modifications (CPLM) [14].

Previous reports indicated that combining exogenous expression of mutated SUMOs, affinity enrichment, and MS technology is a valid approach for identifying sumoylation sites (Figure 4D). According to the sequence of SUMOs, the R92Q mutation in SUMO1 could shorten the remnant amino acid residue after trypsin digestion, which is beneficial for the MS identification of sumoylation sites [132]. By this approach, 17 sumoylation sites on 12 proteins were identified at the amino acid resolution [132]. Similarly, by constructing SUMO1 T95R and SUMO2 T91R variants, 295 SUMO1- and 167 SUMO2-modified sites in human cells were identified, respectively [133]. Sequentially, His-tagged SUMO2 with a lysine deficient (K0) and a Q87R mutation was constructed and stably overexpressed in a HeLa cell line, which led to 4349 SUMO2-modified sites to be identified under SUMO protease inhibition, proteasome inhibition, and heat shock treatment [134]. Based on the similar strategy, a series of methods to identify sumoylation sites were developed, and more sumoylation sites were identified, including 203 SUMO2-modified sites identified in cells expressing a Flag-tagged SUMO2 K0Q87R mutant [135], ∼ 1000 SUMO2-modified sites in cells expressing a His-tagged SUMO2 T90K mutant [136,137], 954 SUMO3-modified sites in cells expressing a His-tagged SUMO3 Q87R/Q88N mutant [138], 976 sumoylation sites in human stem cells expressing a SUMO1/2 mutant [139], and 40,765 SUMO2-modified sites through an enhanced K0-SUMO strategy [140].

Although the approaches based on SUMO mutations have been successfully developed for the identification of sumoylation sites, there are many drawbacks, such as inhibition of poly-sumoylation because of mutation of lysine to arginine. Hendriks et al. developed the protease-reliant identification of SUMO modification (PRISM) method without a SUMO mutation to map the sumoylation sites, which enabled the identification of 751 wild-type sumoylation sites on endogenous proteins, with 200 sites in response to heat shock [141]. Similarly, 25 SUMO3-modifed sites on 23 proteins were identified to be related to senescence by two steps of enrichment [142]. Using sequential peptide immunopurification, 983, 10,388, and more than 8000 sumoylation sites were identified in three individual studies, which revealed the potential substrates of PIAS1 and crosstalk between sumoylation and ubiquitylation [143–145].

Recently, proteomic methods mapping endogenous sumoylation sites without exogenous expression of SUMOs have also made great progress. Lumpkin et al. digested proteins using wild-type α-lytic protease (WaLP), a protease that cleaves proteins after a threonine (T) residue, which leaves the GG residue at the C-terminal of SUMOs recognized by the K-ε-GG antibody [146] (Figure 4E). Through this approach, 1209 endogenous sumoylation sites were identified under completely native conditions [146]. Additionally, by combining different protease digestion and peptide-level immunoprecipitation enrichment strategies, 14,869 endogenous SUMO2/3-modified sites in human cells were identified in response to heat shock and proteasome inhibition, and 1963 sumoylation sites were quantified across 8 mouse tissues [147]. In addition, based on the characteristics of tryptic sumoylated peptides, an antibody-free enrichment method for the identification of endogenous sumoylation sites was also developed, which led to 177 SUMO1-modified sites and 74 SUMO2/3-modified sites being identified [148].

### Acylation

Lysine acylations on histones mainly regulate histone folding and the dissociation of histone octamers, which leads to a change in the structure of nucleosomes and further mediates the transcriptional regulation of gene expression. Lysine acylations on non-histone proteins mainly regulate protein stability, cytoskeleton regulation, organelle localization, enzyme activity, and protein–protein interaction. With the rapid development of antibody technology and MS technology, the types of protein acylations have been expanded from classical simple modification types, such as Kac, Kpr, and Kbu, to more complex modification types such as Kcr, Kmal, and Kglu. More studies have revealed that these diverse modification types are closely related to the occurrence and development of metabolic diseases, cancers, inflammation, and other many diseases. Since the first global Kac proteomics study was reported [149], the study of acylation modifications has greatly expanded our accurate understanding of the histone code, epigenetic regulation, metabolic regulation, and signaling transduction.

#### Hydrophobic acylation group

Kac is the most common type of lysine acylations. However, due to its low cellular abundance and technological limitations during its initial discovery, the progress of Kac research was very slow. Since Kac was first reported on lysine residues of histones, tubulin has been identified as the second acetylated protein in the cell, representing the first non-histone substrate of Kac. In the following years, numerous additional non-histone Kac substrates have been discovered, such as the tumor suppressor p53 protein and the human immunodeficiency virus (HIV) transcriptional regulator Tat protein. With the improvement of antibody-based affinity enrichment and MS technologies, Kac studies have become increasingly more in-depth and efficient. In 2009, 3600 Kac sites on 1750 proteins were reported in a human leukemia cell line by combining technologies of antibody enrichment, high performance liquid chromatography (HPLC) off-line fractionation, and MS, which greatly expanded the range of Kac substrates [150]. In human liver tissues, more than 1000 acetylated proteins were reported [151,152], and it was discovered that Kac generally existed in human metabolic enzymes and further regulated the activities of these metabolic enzymes. Currently, the number of studies on Kac is increasing, and the roles of Kac in diverse cellular activities and various diseases are constantly reported [153]. Our previous study used antibody enrichment, off-line HPLC separation, and MS technology to deeply construct the Kac map in *Staphylococcus aureus* [154]. In total, 1361 Kac sites on 412 proteins were identified, and these acetylated proteins were widely involved in multiple regulatory pathways in cells.

According to the principle of reaction, Kac can be divided into enzymatic reactions and non-enzymatic reactions [155]. It is reported that the lysine side chain in eukaryotes can be directly acetylated by acetyl-CoA, while in prokaryotes the lysine can be directly acetylated by acetylphosphate. To deeply understand the kinetic characteristics of enzymatic acetylation reaction, Choudhary’s group used quantitative proteomics technology to systematically study the specific acetylation catalytic sites of p300/CBP, and deeply constructed the dynamic acetylation turnover map in cells [156].

Kpr has demonstrated a wide range of regulatory functions of non-histone proteins, including but not limited to the modulation of metabolic enzyme activities and metabolic pathway networks [157], and is closely related to spermatogenesis [158]. In addition to mammalian cells, Kpr was also found to be widely present in bacteria. In thermophilic bacteria, 361 Kpr sites were identified, suggesting that the Kpr level is significantly increased in the late period of the bacterial growth cycle [159]. Our previous study systematically identified 1467 Kpr sites on 603 proteins from *Escherichia coli* [160]. We found that the Kpr level in *E. coli* was sensitive to propionate in the environment and that the Kpr level significantly decreased under high glucose conditions, which was in contrast to the change in the Kac level. Our study also revealed that Kpr has a unique role in the metabolism of carbohydrates in bacteria and is regulated by deacetylase CobB and acetyltransferase PatZ. Furthermore, we performed a deep analysis of the Kac and Kpr profiles in *Mycobacterium tuberculosis*, and demonstrated that these two different types of modifications have unique functions in *M*. *tuberculosis* [161].

Kbu was discovered together with Kpr [41]. p300 and CBP catalyze the formation of Kbu on histones *in vivo*, which plays a key role in transcriptional regulation. Currently, most of the research on Kbu focus on histones, with only a limited amount of attention given to non-histone proteins. Several non-histone substrates have been reported to undergo Kbu in mammalian cells. For instance, proteomic data have revealed the presence of three Kbu sites on p53 (K373, K373, and K382), four on p300 (K1554, K1555, K1558, and K1560), and two on CBP (K1595 and K1597). In *Clostridium acetobutylicum*, 1078 Kbu sites on 373 proteins were identified, which constituted a resource for functional study in bacteria [162].

Kcr on histones can be catalyzed by p300 [163], which has an important role in the regulation of male germ cell differentiation [42]. AF9, a YEATS domain-containing protein, is a reader of Kcr [64], first expanding the evolutionally conservative YEATS domain into the crotonyl lysine reader family. In 2017, HDACs were reported as the main histone decrotonylase [63]. CDYL transformed crotonyl-CoA into β-hydrobutyryl-CoA, and then negatively regulated histone Kcr [164]. This negative regulation was intrinsically related to transcriptional repression and participated in the regulation of the reproductive process. Previous studies indicated that Kcr not only occurred on histones, but also on non-histone proteins [165]. Yu et al. systematically identified 14,311 Kcr sites on 3734 proteins in HeLa cells before and after *CDYL* knockout [166], which constructed the largest dataset of Kcr modifications in mammalian cells. Furthermore, this study revealed the important role of CDYL in the regulation of homologous recombination by mediating Kcr of the non-histone protein RPA1. Similarly, a Kcr map of cardiac tissue after ischemia/reperfusion injury was also constructed, suggesting that Kcr plays a cardiac repair function by regulating mitochondrial proteins and myofilament proteins [167].

Sodium benzoate is widely used as a food additive and has been approved by FDA for treating hyperammonemia caused by a urea circulation disorder. In 2018, benzoylation on histones was discovered through MS and biochemistry methods [45]. A total of 22 Kbz sites on histones were identified. The Kbz level was affected by exogenous sodium benzoate and regulated by the SIRT2 enzyme *in vivo*. A study demonstrated that the DPF family and YEATS family proteins are readers of histone Kbz [68]. Kbz also occurs on non-histone proteins. Through proteomics technology, 207 Kbz sites on 149 non-histone proteins were identified [67], which are involved in ribosome biosynthesis, glycolysis, gluconeogenesis, and ribosomal RNA processing. This study revealed that Gcn5 is a histone benzoyltransferase in yeast, Hst2 is one of the lysine benzoylases and has no specificity for sequence selection, and Taf14 and Sas5 are the potential readers of Kbz.

Long-chain fatty acylations like myristoylation (14-carbon) and palmitoylation (16-carbon) on lysine residues are known as lysine fatty acylation (KFA) [168]. Their reported substrates and regulatory enzymes were well summarized in recent reviews [169]. Current proteomics analysis of KFA is mainly dependent on clickable fatty acid analogs that metabolically label and enrich KFA substrates. In 2018, Liu et al. discovered that IcsB was an 18-carbon fatty acyltransferase critical for survival and pathogenesis of intracellular Gram-negative bacterium *Shigella flexneri* [170]. Chemical proteomics profiling discovered about 60 substrates of IcsB, most of which were membrane-associated proteins like Rho GTPase and CHMP5. IcsB modified Rho GTPase with 18-carbon fatty acyl groups and disrupted its membrane cycling. IcsB also modified CHMP5 and enhanced *S*. *flexneri* escape from autophagy. In 2019, Cao et al. identified HDAC11 as a novel lysine defatty-acylase, and further profiled HDAC11 substrates by combining SILAC-based quantitation with chemical proteomics platform [171]. They found that serine hydroxymethyltransferase (SHMT) was defatty-acylated by HDAC11, which further regulated the stability of type 1 interferon (IFN) receptor and type I IFN signaling.

#### Polar acylation group

Khib was first discovered on histones in 2014 [69], and Khib of histones has been shown to play a crucial regulatory role in the differentiation of sperm cells. Currently, Khib has been reported to be ubiquitous on non-histone proteins, spanning from prokaryote to mammal. By utilizing proteomics technology, 4735 Khib sites on 1051 proteins in *Proteus mirabilis* were identified [172]. These Khib-modified proteins were involved in various metabolic pathways, such as purine metabolism, pentose phosphate pathway, and glycolysis/gluconeogenesis, indicating a ubiquitous prevalence of this alteration in prokaryotic cells. Additionally, 1458 Khib sites on 369 proteins in yeast cells were also identified, highlighting the crucial role of Khib in regulating sugar balance within eukaryotic cells [173]. Meanwhile, by using proteomic methods, the researchers identified 7937 Khib sites on 1901 proteins in HeLa cells, revealing TIP60’s non-acetylated transferase function and demonstrating its role as a histone 2-hydroxyisobutyryltransferase [73].

CobB has been reported as an eraser for Khib removal in prokaryotic *E*. *coli*, facilitating the regulation of metabolic enzyme activity and altering the molecular mechanism of cell growth [174]. Meanwhile, acetyltransferase TmcA was identified as a writer to catalyze Khib in *E*. *coli*, which can mediate transcriptional regulation and enhance bacterial acid resistance [175]. The acetyltransferase EP300 has been reported to not only regulate protein Kac levels, but also directly and effectively catalyze Khib. Using the SILAC-based quantitative proteomics technique, a total of 4239 Khib sites were identified, 149 of which were significantly regulated by EP300 [72]. EP300-regulated Kac is typically associated with RNA-related biological functions, but EP300-regulated Khib is closely linked to glycolysis, carbon metabolism, and amino acid biosynthesis, which is different from the Kac modification profiles.

A recent study revealed the crucial role of Khib in tumor invasion and metastasis [176]. The researchers constructed the Khib landscape of primary/metastatic ESCC tumor tissue. A total of 5384 proteins and 39,997 Khib sites were quantified. Among the Khib sites, NAT10 K823hib was significantly up-regulated in transferred tissues. Additionally, the researchers revealed that KAT7 acted as the writer to catalyze Khib on NAT10 K823 while SIRT7 functioned as the eraser for Khib removal. Furthermore, the deubiquitylation enzyme USP39 was found to be recruited by NAT10 K823hib to enhance the stability of NAT10, leading to the up-regulated expression of NAT10. This study revealed that Khib enhanced the stability of NAT10 and promoted tumor invasion and metastasis.

Kbhb was mainly enriched in the promoter region of the activated gene and mediated the metabolic regulation of the hunger response [70]. β-hydroxybutyric acid is an intermediate metabolite of the fatty acid oxidation process and also one of the main components of ketone bodies, which could mediate histone Kbhb. The non-histone protein, tumor suppressor protein p53, was reported to be β-hydroxybutyrylated through the CBP enzyme [177], which led to a significant reduction in its Kac level and activity and promoted the growth of cancer cells. In addition, 3248 Kbhb sites on 1397 proteins were identified in HEK293 cells [178]. A previous study indicated that Kbhb directly inhibited the activity of AHCY, a key rate-limiting enzyme in the methionine cycle, thereby regulating one-carbon metabolism in tumor cells [179]. In all, Kbhb performs a diverse array of functions in various cellular processes, including chromatin remodeling and transcriptional regulation, and mediates the occurrence and metastasis of tumors [180].

Lactic acid is a byproduct of cellular glucose metabolism. For a long time, lactic acid was regarded as waste produced during glycolysis. In 2019, Kla was discovered as a new lysine modification on histones [71]. This study found that Kla participated in the steady-state regulation of M1 macrophages infected by bacteria. More recently, studies showed the importance of protein Kla in functions related to glycolysis, macrophage polarization, nervous system regulation, and other important processes. In cells, the expression of glycolysis-related genes was regulated by transcription factor Glis1 [181]. The activation of glycolysis-related genes led to an increased glycolysis level, which further increased the Kla level on histones and promoted transcription. Notably, the level of Kla in ocular melanoma samples was higher than that in normal samples, which revealed that the lactylation of histone H3K18 in ocular melanoma had an import role in tumorigenicity [182]. Lactic acid promoted the Kla of HMGB1 through a p300/CBP-dependent mechanism, and the balance of Kla and Kac on HMGB1 in macrophages promoted the development of polymicrobial sepsis [183]. Kla was reported to regulate the ubiquitin–proteasome system in systemic lupus erythematosus (SLE) pathogenesis, which preceded the autophagic removal of mitochondria [184]. Kla was also reported to be present in brain tissue and regulated the nerve excitation and behavior-related stimuli [185]. The long-term excessive intake of lactic acid by neurons can cause peripheral nerve damage [186]. To improve the identification of the Kla level, a recent study found that lysine-lactylated peptides generated the cyclic imine ion under high energy collision fragmentation when detected by MS [187]. Based on this characteristic ion, the researchers firstly analyzed the large-scale Kla substrates from public human proteome data. They discovered that Kla was highly enriched in the glycolysis pathway. The accumulation of lactic acid within cells led to Kla on metabolic enzymes involved in the glycolysis pathway, thereby exerting negative feedback regulation on the glycolysis pathway.

#### Acidic acylation group

Ksucc is a key lysine acylation with an acidic group. SIRT5 is a major desuccinylase in cells [84]. Our previous study systematically compared the global changes of Ksucc in mammalian cells before and after *SIRT5* knockout, and identified 2565 Ksucc sites on 779 proteins, most of which did not coincide with Kac sites, suggesting that Ksucc has a unique function in cells. Our study revealed that Ksucc played an important role in the regulation of cell metabolism [188]. In 2013, we reported 2580 Ksucc sites on 670 proteins in *E. coli* and found that the Ksucc level was higher than the Kac level under high glucose conditions. Our study also revealed CobB as a desuccinylation enzyme in a prokaryotic cell [189]. Recent studies revealed that Ksucc has an important role in regulating the occurrence and development of tumors, subarachnoid hemorrhage (SAH), and other diseases. For example, K311succ on the mitochondrial protein glutaminase (GLS) in human pancreatic ductal adenocarcinoma (PDAC) tissues enhanced the catabolism of glutamine, and promoted the production of nicotinamide adenine dinucleotide phosphate (NADPH) and glutathione, thus counteracting oxidative stress and promoting the tumor growth [190]. Ksucc was reported to be extensively present in the brain tissues of mice with SAH, and most of the succinylated proteins were located in mitochondria [191]. The reduced expression of SIRT5 in the brain tissues of SAH patients led to elevated Ksucc level, leading to mitochondrial metabolic dysfunction and impacting neural cell activity.

Kmal was identified as the new type of lysine PTM on histones through SILAC-based high-throughput quantitative proteomics and antibody-based affinity enrichment technology [80]. Moreover, SIRT5 was found to catalyze lysine demalonylation reactions both *in vitro* and *in vivo* [80]. In a deep Kmal analysis on *SIRT5*-knockout and wild-type mouse liver tissues, 1137 Kmal sites on 430 proteins were identified [192]. They found that the glycolytic pathway was significantly inhibited in *SIRT5*-knockout mice. In the liver tissues of type 2 diabetic and control mice, more than 200 malonylated proteins were identified, about 70% of which were closely related to the metabolic pathways of sugars and lipids [193]. This study also demonstrated a significant increase in Kmal levels within the liver tissue of type 2 diabetic mice compared to control mice, indicating that Kmal played an important role in type 2 diabetes. Our previous study used SILAC-based quantitative proteomics technology to systematically quantify 1745 Kmal sites on 594 proteins in *E. coli* and found that Kmal was closely related to fatty acid metabolism [194]. We found that Kmal regulated the activity of citrate synthase, one of the rate-limiting enzymes of the TCA cycle. We also systematically studied the effect of other acylation modifications, such as Kac and Kbu, on the biosynthesis of microbial secondary metabolites [157,195]. Our results indicated that the accumulation of high concentrations of acyl-CoA in bacteria promoted the production of secondary metabolites. However, the high concentration of acyl-CoA also triggerred the feedback regulation through acyl-CoA modification, which inhibited the key enzymes involved in secondary metabolism and impacted the synthesis of these metabolites.

Biotinylation is a conserved lysine PTM among species [196]. Biotin-dependent carboxylases, such as acetyl-CoA carboxylase (ACC) in bacteria and acetyl-CoA carboxylases 1 and 2 (ACC-1 and ACC-2), propionyl-CoA carboxylase (PCC), pyruvate carboxylase (PC), and methylcrotonyl-CoA carboxylase (MCC) in human, are convinced biotinylation substrates[197]. Biotin is covalently attached to a specific lysine residue located in a Met–Lys–Met sequence by BirA in bacterial and holocarboxylase synthetase (HCS) in mammalian. Biotin is released by biotinidase after those carboxylases are hydrolyzed and reutilized for biotinylation of newly synthesized carboxylases. Beyond those carboxylases, several studies proposed that histones were also biotinylation substrates of HCS and histone biotinylation served as an epigenetic marker [198,199]. However, the existence of histone biotinylation was disputed as researchers doubted the specificity of Western bolt analysis by streptavidin and antibody [200]. Moreover, in MS analysis of affinity-enriched biotinylated peptides, histone biotinylation was undetectable in native cells. Up to now, whether biotinylation occurs on other substrates and plays function beyond carboxylation remains largely unknown.

In addition to these common lysine PTMs, other types of PTMs on lysine residues, such as glycation and polyphosphorylation, have also been reported [201,202]. These additional lysine PTMs are also implicated in various cellular processes by regulating the biological functions of both histone and non-histone proteins. However, due to technological limitations, the system-wide analysis of these lysine PTMs is still full of challenges.

## Lysine PTM crosstalk

PTM crosstalk, also known as the combinational action of PTMs, is a regulatory mechanism in which one PTM affects the occurrence of other PTMs, and is a specific and sophisticated molecular mechanism involved in numerous biological processes, such as gene expression, signal transduction, and protein degradation. Previous reports classified PTM crosstalk into positive and negative types or intra- and inter-protein forms based on functions or positions [12,203]. As stated previously, more than 30 types of PTMs occur on lysine residues, and about 300,000 modified sites have been identified according to the CPLM database [14]. Notably, of these modified lysine residues, more than 150,000 lysine residues can have two or more types of PTMs, which represents a complicated molecular mechanism in physiology and pathology.

### Histone PTM crosstalk

**Figure 5:**
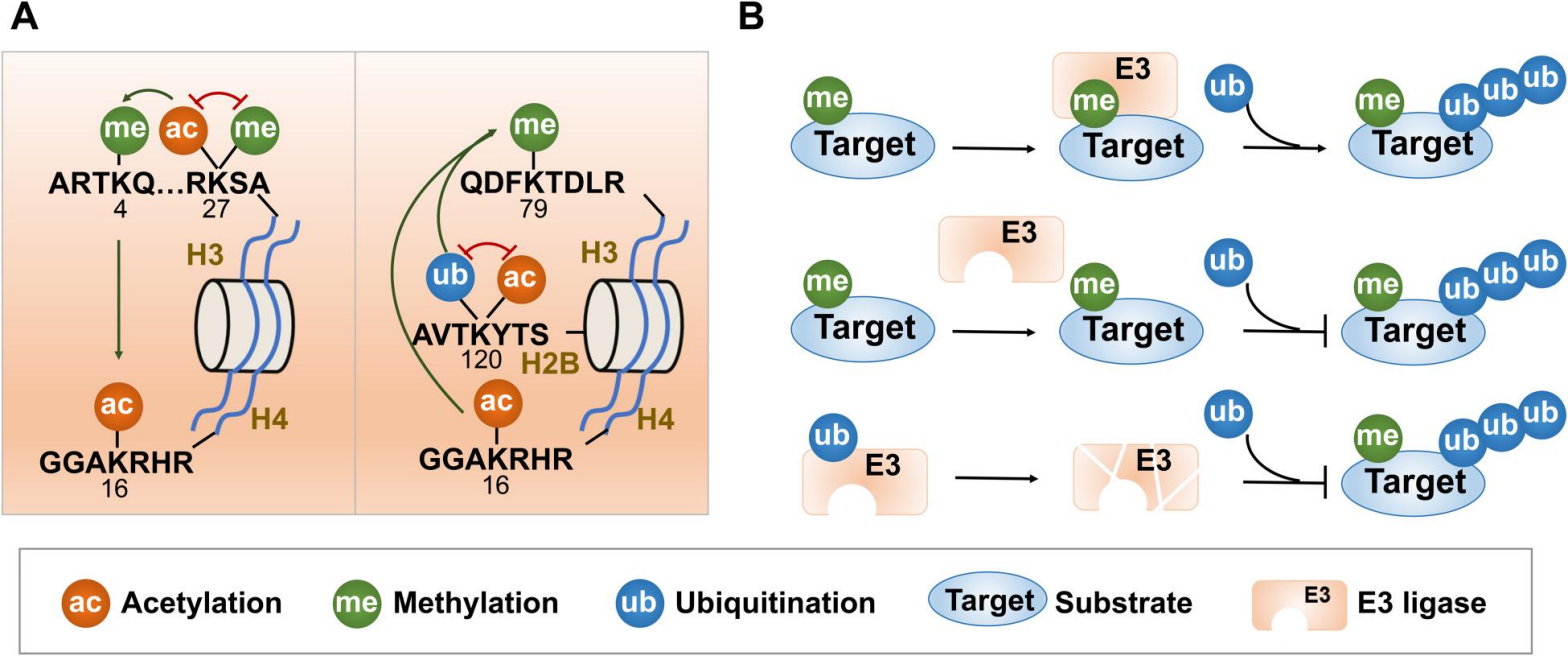
Examples of lysine PTM crosstalk on histone and non-histone proteins **A**. Examples of lysine PTM crosstalk on histone. Left: methylation and acetylation on H3K27 compete each other, acetylation on H3K27 enhances the level of methylation on H3K4, and methylation on H3K4 promotes the acetylation on H4K16. Right: ubiquitination and acetylation on H2BK120 compete each other, and co-occurrence of ubiquitination on H2BK120 and acetylation on H4K16 enhances the methylation on H3K79. **B**. Examples of crosstalk between lysine ubiquitination and other lysine PTMs on non-histone proteins. Upper: methyl-dependent ubiquitination. Middle: competition between methylation and ubiquitination at the same lysine site. Lower: auto-ubiquitination of E3 ligases regulates the ubiquitination of substrates. PTM, post-translational modification.

Histones are subjected to diverse PTMs. In many cases, PTMs on histones usually co-occur and co-regulate nucleosome structure and dynamics in an integrated manner. Previous studies proposed that PTM crosstalk was a specific grammar of the complex language of histone PTMs for modulating the chromatin state and transcriptional activity. Reciprocal interactions between Kac and Kme on histones, such as H3K4me and H4K16ac, were also taken as a classic example to illustrate the regulatory mechanism of histone PTM crosstalk [204]. In addition, H3K27ac in the promoter region increased the H3K4me3 level around transcriptional start sites and activated transcription [205]. Our study also indicated that crosstalk between lysine acetylation (H3K27ac) and methylation (H3K27me) affected the pharmacological activity of the EZH2 inhibitor on solid tumors, which provided a combination therapy strategy for drug-resistant tumors [206] (**Figure  5**A, left panel; Table S2).

Accumulating evidence indicated that ubiquitination-dependent histone crosstalk plays a vital role in the orchestrated regulation of chromatin as well as DNA repair [207,208]. A previous report reviewed a crucial type of crosstalk between Kub and other PTMs including sumoylation, Kac, and Kme on histones during DNA DSB repair [209]. Recent studies further showed that the reduction of KAT5-mediated H2BK120ac increased the ubiquitination level of K120 on H2B (H2BK120ub), and this acetylation–ubiquitination switch signaling regulated repair of DSBs by homologous recombination [210] (Figure 5A, right panel). The monoubiquitination at K13 and K15 on H2A inhibited the formation of the polyubiquitination chain and simultaneously promoted ubiquitination at the C terminal of H2A, which is also a crucial mechanism for DNA repair [211]. In addition, the crosstalk between H2BK120ub and H3K79me is a well-characterized example of how Kub affects the chromatin process. The structural analysis confirmed that the H4 tail was vital for the promotion effect of H2BK120ub on H3K79me [35]. Furthermore, Valencia-Sánchez et al. found that the co-occurrence of H4K16ac and H2BK120ub enhanced the level of H3K79me by restricting the search space of continuously activated DOT1 methyltransferase on chromatin, which regulated gene transcription by orchestrating the chromatin state [212] (Figure 5A, right panel). The crosstalk among H4K16ac, H2BK120ub, and H3K79me is helpful for us to understand the crucial role of the DOT family methyltransferase in tumorigenesis. In addition to ubiquitination-dependent direct regulatory mechanisms, a recent study discovered that the ubiquitination at K34 on H2B indirectly interacted with DOT1L to catalyze H3K79me by mediating histone distortion, suggesting a novel regulatory mechanism of histone crosstalk [213]. Other types of ubiquitination-dependent crosstalk are equally crucial for diverse functions. For example, H2AK119ub regulated transcription activity by enhancing the levels of H3K4me3, H3K27me3, and H3K36me3 [30,31]. Polyubiquitination of H3K79 mediated by CRL4^DCAF8^ E3 ligase promoted H3K9 methylation and repressed transcription of fetal liver genes [214]. H2BK120ub increased the level of H3K4me3 in a gene-specific manner during memory formation [36].

Similarly, other ubiquitin-like modifications, such as neddylation and sumoylation, also affect the chromatin process by crosstalking with other PTMs. For example, polyneddylation at the N-terminal lysine residues of H4 activated a DNA damage-induced Kub cascade by promoting the recruitment of the E3 ligase RNF168 at DNA damage sites, facilitating DNA damage repair [215]. H4K12 sumoylation repressed the levels of H4 tail acetylation and H3K4 methylation and drove the silencing of regulated genes [216].

In addition, links between the same types of PTMs are also crucial histone PTM crosstalk. For example, both H3K4me3 and H3K36me2/3 hindered H3K27me3 by inhibiting the activity of PRC2 to activate transcription [217]. Loss of SETD2-mediated H3K36me3 elevated H3K79me3 to differentially regulate tumor suppressors and oncogenes, which accelerated mixed-lineage leukemia (MLL)-rearranged leukemogenesis [218].

### Non-histone PTM crosstalk

Similar to histone PTM crosstalk, PTMs on non-histone proteins can also modulate other PTMs to regulate protein functions. However, previous studies indicated that lysine PTM crosstalk on non-histone proteins plays distinct roles in regulating downstream biological effects.

PTM crosstalk-mediated protein stability is a crucial mechanism for maintaining cellular homeostasis. A well-studied example is phosphorylation-dependent protein ubiquitination and degradation, which represents a typical mechanism of intra-protein PTM crosstalk on non-histone proteins for modulating protein stability [219,220]. Similarly, lysine PTMs can also promote protein degradation by crosstalking with ubiquitination. For example, Kme regulates protein stability by forming methylated degron recognized by E3 ligases in a ubiquitination-dependent manner [221] (Figure 5B, upper panel; Table S2). One study reported that EZH2-mediated mono-methylation of RORa at K38 promoted ubiquitination and degradation by recruiting E3 ligase DCAF1, which created the concept of methyl degron-dependent protein degradation [221]. Recent studies to confirm this mechanism reported that DNA methyltransferase DNMT1, E3 ubiquitin ligase UHRF1, hypoxia inducible factor HIF1α, peroxisome proliferator-activated receptor γ coactivator-1α (PGC-1α), and transcription factors E2F1 and SOX2 could also be ubiquitinated and degraded in a methylation-dependent manner [222–226]. In addition to Kme, other PTMs can also trigger protein degradation in a ubiquitin-dependent manner. For instance, acetylation of MPP8 at K439 and of GS at K11 and K14, and sumoylation of FXR at K325 and of c-Myc also induced ubiquitin-dependent protein degradation by ubiquitin–proteosome system [227–230]. While accumulating evidence has consolidated this interesting mechanism mediated by PTM crosstalk, the regularity of Kme, Kac, or other PTM degrons that regulate protein stability remains unknown.

Conversely, since Kub and other lysine PTMs usually occur at the same sites, lysine PTMs could disrupt ubiquitin-dependent protein degradation by competing with each other (Figure 5B, middle panel). For example, Kac of histone demethylase JMJD1A, ATP-citrate lyase ACLY, DNA-binding protein hSSB1, poly(ADP-ribose) polymerase PARP1, transcription factor Snail, splicing factor SRSF5, tumor suppressor p53, and E3 ligase murine double minute 2 (MDM2) blocked their degradation by inhibiting Kub [231–238]. Furthermore, previous studies found that sumoylation of KDM5B, TWIST2, PES1, and MRE11 protected them from ubiquitin-mediated protein degradation, suggesting that diverse types of lysine PTMs regulate protein stability by crosstalking with Kub [239–242].

In addition, other types of intra-protein crosstalk among lysine PTMs were also reported. For example, the sumoylation of p53 at K386 was inhibited following p300-mediated acetylation [243]. A recent study reported the methylation-dependent sumoylation motif [244], which provides a new clue for exploring the crosstalk between methylation and sumoylation.

Inter-protein PTM crosstalk is that modifications on one protein regulate modifications of another protein to influence downstream signals. PTM crosstalk between PTM regulatory enzymes and substrates is a common inter-protein PTM crosstalk. An example is an interplay between neddylation and ubiquitination. Neddylation of cullin proteins, the scaffold of cullin-RING E3 ubiquitin ligases, is indispensable for activating CRL family E3 ligases, which positively regulates much of protein turnover by ubiquitin–proteasome system [108,245,246]. In addition, auto-modification of PTM regulatory enzymes, a mechanism that PTM regulatory enzymes modify their own modification, can also create crosstalk by modulating downstream substrates. For example, auto-methylation of EZH2 increased the Kme level of substrates by enhancing the activity of EZH2 [247,248]. Auto-acetylation of p300 enhanced its catalytic activity, which might affect the level of diverse acylation modifications because of its multiple roles in regulating acylation [249]. Notably, auto-ubiquitination of the E3 ligase MDM2 not only enhanced its ubiquitin ligase activity to promote substrates for degradation but also stabilized substrate proteins by accelerating its own degradation [250,251] (Figure 5B, lower panel).

Accumulating evidence indicates that lysine PTM crosstalk occurs across the proteome. Nevertheless, the types of lysine PTM crosstalk are still only sporadically reported because of the limitation of technologies. State-of-the-art MS technology can identify and quantify diverse types of PTMs at the single amino acid resolution, which provides an essential tool for uncovering the global picture of lysine PTM crosstalk [12,252].

## Druggable targets of lysine PTM regulators

Lysine PTMs are involved in diverse cellular functions, which are orchestrated by various PTM regulatory factors including writers, erasers, and readers. Dysregulation of lysine PTMs mediated by these factors is closely related to the occurrence and development of diseases. Thus, targeting lysine PTM regulators has been a critical strategy for disease therapy. Currently, over 20 lysine PTM regulators have been approved as drug targets by the U.S. Food and Drug Administration (FDA) or are in clinical trials. For these druggable targets, five drugs targeting Kac, one drug targeting Kme, and six drugs targeting ubiquitin-like modifications have been approved by the FDA for cancer treatment (Table S3).

### Writers

The enzymes catalyzing PTMs at specific amino acids are known as PTM writers. The enzyme p300/CBP is a lysine acyltransferase (KAT), which catalyzes Kac, Kpr, Kbu, Kcr, Khib, and Kbhb. Previous studies indicated that p300/CBP promoted the proliferation and metastasis of several cancers including liver cancer, non-small cell lung cancer, prostate cancer, melanoma, and hematological malignancies [253–257]. Thus, p300/CBP has been used as a potential drug target for cancer therapy, and two inhibitors targeting p300/CBP are in the clinical trials for tumor therapy [254,257]. Similarly, lysine methyltransferases, including EZH2, DOT1L, G9A, and SUV39H1, catalyze Kme, and are also the clinical drug targets for cancer treatment [258–261]. Among them, tazemetostat, an EZH2 inhibitor, has been approved by the FDA for the treatment of sarcoma and follicular lymphoma [262,263].

In contrast to Kac and Kme, ubiquitin-like modifications including Kub, sumoylation, and neddylation are orchestrated by E1, E2, and E3 enzymes. Of these three types of enzymes, NEDD8-activating enzyme (NAE) is a type of E1 enzyme that activates the NEDD8 protein to initiate protein neddylation, which is associated with various types of cancer [264]. The specific inhibitor targeting NAE, MLN4924, has been in phase III clinical studies for hematological malignancies including acute myeloid leukemia, chronic myeloid leukemia, and myelodysplastic syndrome [265]. Notably, E3 ligases confer the specificity of protein substrates, and their aberrant expression directly affects the stability of substrates. MDM2 is a crucial regulator of the tumor suppressor protein p53, and MDM2 overexpression leads to the degradation of p53 and promotes the survival of cancer cells [250]. The inhibition of MDM2 can significantly repress the proliferation of cancer cells, and eight inhibitors targeting MDM2 have been developed for several solid tumors and hematological malignancies [266]. More strikingly, targeting E3 ligases to induce protein degradation is an attractive approach for “undruggable proteins”. Among about 600 E3 ligases, VHL, CRBN, and DCAF15 are valid drug targets for protein degradation. For example, immunomodulatory drugs, including thalidomide, lenalidomide, and pomalidomide, specifically induce protein degradation and display anti-cancer effects by targeting CRBN [115,267,268]. Similarly, the aryl sulfonamide agents, indisulam, chloroquinoxaline sulfonamide (CQS), and tasisulam, promote protein degradation by binding to DCAF15 [269]. Recently, based on the same mechanisms, some small-molecule inhibitors based on proteolysis-targeting chimeras (PROTACs) are developed for cancer therapy [11].

### Erasers

Erasers are the enzymes that remove PTMs from substrates. Accumulating evidence suggests that some erasers regulating lysine acylation, Kme, and ubiquitin-like modifications are biomarkers related to tumor prognosis and thus are important targets of drug development. HDACs function as deacylases to maintain the intracellular acylation levels. Overexpression and mutation of HDACs occurred in diverse cancers [10]. Five inhibitors targeting HDACs have been approved for multiple myeloma, T-cell lymphomas, peripheral T cell lymphomas, and breast cancer, and many HDAC inhibitors are in clinical trials for various cancers [10]. In addition, the inhibitors targeting two lysine demethylases, LSD1 and KDM5B, are also in clinical trials [9]. DUBs regulate the Kub level of protein substrates to maintain protein stability, and their dysregulation leads to an imbalance of cellular homeostasis [8]. DUBs have two different effects on mediating protein stability. DUBs in the proteasome promote protein degradation by removing ubiquitin chains, but conversely, other DUBs suppress the degradation of protein substrates which are not recognized by proteasome through detaching ubiquitination chains [8,270]. Accumulating evidence showed that DUBs are an important cause of tumorigenesis. Currently, several drugs targeting DUBs are in the preclinical phase, suggesting that targeting DUBs might be a new approach for cancer therapy [7].

### Readers

Readers recognize and bind protein PTMs to regulate protein function or gene expression. Overexpression, translocations, and mutations of readers can drive tumorigeneses. Recently, readers for Kac, Kme, and Kub have become important targets, for which inhibitors have been developed for cancer therapy. BET proteins were important readers of acetylated proteins to regulate gene expression and were implicated in tumorigenesis. BET protein inhibitors including I-BET762, CPI-0610, and TEN-010, as well as the Kub reader p97/VCP inhibitor CB-5083, are in clinical trials for several solid tumors and hematological malignancies [6,271,272].

The proteasome is a complex that recognizes and degrades ubiquitinated substrates, and is a drug target approved by FDA for cancers [273,274]. The proteasome consists of multiple proteins including ubiquitination-recognizing proteins, ubiquitination-removal erasers, and protein-degraded enzymes [275]. Currently, three drugs targeting the proteasome have been approved for hematological malignancies, and three drugs are in the clinical phase.

## Conclusion and perspectives

Lysine PTMs are important types of protein PTMs that regulate the functions of histone and non-histone proteins. With the development of antibody-based affinity enrichment technology coupled with MS technology, the discovery rate of novel types of PTMs has been increasingly. Currently, over 30 types of lysine PTMs are reported, and ∼ 300,000 lysine PTM sites have been identified. Nevertheless, whether other new lysine PTMs exist is unknown, and how to achieve the identification and quantification of globally modified sites is still a challenge.

PTM crosstalk is a nuanced and sophisticated molecular mechanism to regulate protein functions, serving as a specific language for protein and other molecular communications. Several types of lysine PTM crosstalks, such as the Kac amd Kme crosstalk and the Kme and Kub crosstalk, have been sporadically reported, and several functions of these lysine PTM crosstalks have been revealed. More importantly, the crosstalk between lysine PTMs and other PTMs, such as phosphorylation and ubiquitination, also plays a crucial role in the regulation of protein functions. However, the new types of PTMs and the global picture of the biological functions of lysine PTM crosstalks are by far unexplored, and the codes of these specific languages need be further deciphered.

Protein PTM regulatory enzymes are important drug targets for disease therapy. Targeting the regulatory enzymes of Kme, Kac, and Kub has been successfully applied to the treatment of diverse diseases. Thus, deciphering the relationship between enzymes and PTMs and characterizing the features of PTMs help reveal potential new drug targets.

Lysine PTMs are evolutionarily conserved across different species spanning from prokaryote to eukaryote, indicating that many lysine PTMs are of ancient origin [276]. With the development of MS coupled with antibody-based affinity enrichment technology, PTM substrates were increasingly identified in different organisms, which provides the opportunity to investigate the conservation and divergence of lysine PTMs across species. A classic example is the conservation of histone modifications regulating gene expression in eukaryote, such as acylation and Kme [277]. For global lysine PTMs, our previous study explored the Kme sites in three species including *E*. *coli*, *Saccharomyces cerevisia*, and HeLa cells by employing MS-based proteomics approach, revealing that methylated proteins were mainly involved in central metabolism in *E*. *coli* and *S*. *cerevisiae*, while were enriched in spliceosome in HeLa cells [278]. By comparing the acetylomes of baker’s yeast and the three deadliest human fungal pathogens, *Cryptococcus neoformans*, *Candida albicans*, and *Aspergillus fumigatus*, Li et al. characterized dramatic evolutionary dynamics and limited conservation of Kac in core biological processes, such as protein translation, histone modifications, TCA cycle [279]. However, due to the diverse types and functions of PTMs in different organisms, it remains a challenging task to develop new computational methods for system-wide analysis of the evolution of PTMs and their crosstalks across different species. Current understanding on this topic is by far limited. Currently, several PTM databases have been constructed for the identified lysine PTM sites and functions, such as CPLM, PhosphoSitePlus, PTMcode 2, and dbPTM (Table S4). Based on PTM evolutionary information, a series of computational predictors have been developed to predict potential lysine PTM sites. For example, KhibPred was developed as a promising computational tool to predict Khib sites based on machine learning frameworks [280]. PSSM-Suc employed evolutionary information of amino acids for predicting Ksucc sites [281]. BiPepGlut used a bi-peptide-based evolutionary method for feature extraction to build Kglu prediction model [282]. SEMal was used to predict potential Kmal sites [283].

In conclusion, lysine PTMs are widespread and versatile protein PTMs, and interpretation of the molecular characteristics and biological functions of lysine PTMs in various physiological and pathological states is beneficial to the understanding of the fundamental life processes as well as new drug discovery.

## CRediT authorship statement

**Bingbing Hao:** Writing – original draft, Visualization, Investigation, Writing – review & editing. **Kaifeng Chen:** Writing – original draft, Visualization, Investigation, Writing – review & editing. **Linhui Zhai:** Writing – original draft, Investigation, Writing – review & editing. **Muyin Liu:** Investigation. **Bin Liu:** Investigation. **Minjia Tan:** Conceptualization, Writing – review & editing. All authors have read and approved the final manuscript.

## Supplementary material

Supplementary material is available at *Genomics, Proteomics & Bioinformatics* online (https://doi.org/10.1093/gpbjnl/qzae019).

## Competing interests

The authors have declared no competing interests.

## Supplementary Material

qzae019_Supplementary_Data
